# Study on clinical characteristics of event‐related potential P300 in elderly schizophrenics and associated risk factors

**DOI:** 10.1002/brb3.2966

**Published:** 2023-04-10

**Authors:** Zhenguo Wu, Zixuan Zhou, Wenting Lu, Lin Liu, Qifeng Zhu, Guanli Su

**Affiliations:** ^1^ Department of Psychiatry The First Hospital of Hebei Medical University Shijiazhuang China; ^2^ Mental Health Institute of the Hebei Medical University Shijiazhuang China

**Keywords:** clinical characteristic, different levels of violence, elderly schizophrenics, event‐related potential P300, risk factor

## Abstract

**Objective:**

To investigate the clinical characteristics of event‐related potential P300 in elderly schizophrenics with different levels of violence and the risk factors of severe violence.

**Methods:**

A total of 138 elderly schizophrenic patients from January 2020 to December 2021 in the First Hospital of Hebei Medical University were enrolled in this retrospective analysis. Based on the violence risk assessment, 61, 102, and 145 patients were divided into high‐risk, medium‐risk, and low‐risk groups, respectively. Clinical characteristics, P300 latency, and P300 amplitude were compared among the three groups followed by a logistic regression analysis of elderly schizophrenics with severe violence.

**Results:**

The latency of P300 in the high‐risk group was higher than that in the low‐risk group (*p* < .05). The P300 amplitude of patients in the high‐risk group was significantly lower than that in the low‐risk group (*p* < .05). Univariate logistic regression analysis showed that previous history of violence, delusion of persecution, P300 latency, and amplitude were independent influencing factors of severe violence in elderly schizophrenics (odds ratio [OR]: 0.022, 95% confidence interval [CI]: 0.007–0.067, *p* < .001; OR: 0.118, 95% CI: 0.043–1.763, *p* = .037; OR: 1.289, 95% CI: 1.142–1.673, *p* < .001; and OR: 0.049, 95% CI: 0.021–0.067, *p* < 0.001, respectively). After adjusting gender, age, and other confounding factors, multivariate logistic regression analysis showed that delusion of persecution, P300 latency, and P300 amplitude were associated with severe violence in elderly schizophrenics (OR: 2.211, 95% CI: 0.061–4.067, *p* < .001; OR: 2.006, 95% CI: 1.421–2.721, *p* = .017; and OR: 0.067, 95% CI: 0.037–0.276; *p* < .001; respectively).

**Conclusion:**

The latency and amplitude of P300 can be used as effective neuroelectrophysiological indicators to evaluate the violence level of elderly schizophrenics. Delusion of persecution, P300 latency, and P300 amplitude were independent influencing factors of severe violence in elderly schizophrenics.

## Graphical abstract

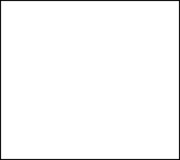

## 1 INTRODUCTION

Based on the Statistics of the World Health Organization, there are currently about 23 million people worldwide suffering from schizophrenia (Mccutcheon et al., 2020). The data from the Mental Health Center of China Center for Disease Control demonstrated that the number of various kinds of mental patients in China has exceeded 100 million, including more than 6.4 million schizophrenics. Mental disorders rank first in the total burden of diseases in China, accounting for about one fifth of the total burden of disease (Zhang et al., 2022). Schizophrenia accompanied by violent behavior refers to the sudden violent behavior of individuals with schizophrenia, including harm or even death directed at themselves or others, and a series of impulsive behaviors directed at property, which do great harm to family and society (Zhang et al., 2021). In recent years, with the aging trend of China's population, the incidence of elderly schizophrenics with violent behavior has been increasing year by year (Li et al., 2020). Therefore, effective methods should be used to predict and judge the violent behavior of elderly schizophrenics.

Previous studies have pointed out that the three main causes of aggression in schizophrenia patients are psychotic symptoms, psychological factors, and impulsiveness (Jauhar et al., 2022). Other studies have confirmed that patients with schizophrenia have cognitive deficits and negative emotion regulation disorders when violent behaviors occur (Richetto & Meyer, 2021). Dominated by symptoms of psychotic diseases, the two can influence each other in a certain stressful situation, which may increase impulsivity and anger, and induce violent events. Event‐related potential (ERP) P300 component is a relatively objective electroencephalogram biological indicator reflecting cognitive psychological activity, which can be used to predict the development of schizophrenia in a susceptible population (Higuchi et al., 2021).

In a related study of male schizophrenia patients, ERP P300 was studied and it was found that the aggressive group had more severe cognitive impairment than the nonaggressive group (Bochkarev et al., 2020). The most prominent manifestation of violence in schizophrenia patients is the prolonged latency of ERP P300 (Bochkarev et al., 2020). However, there are few studies on the clinical characteristics and risk factors of ERP P300 in elderly schizophrenics with different levels of violence.

Herein, this study aimed to investigate the clinical characteristics of ERP P300 in elderly schizophrenics with different levels of violence and risk factors of severe violence, providing favorable evidence for improving the prediction of aggressive behavior.

## 2 METHODS AND MATERIALS

This study was designed as a retrospective study. The study followed the tenets of the Declaration of Helsinki and was approved by the Ethics Committee of the First Hospital of Hebei Medical University.

### 2.1 Subjects

Patients diagnosed with elderly schizophrenia in the First Hospital of Hebei Medical University were recruited from January 2020 to December 2021. The inclusion criteria were as follows: (1) meeting the diagnostic criteria for schizophrenia in the Diagnostic and Statistical Manual of Mental Disorders Fifth Edition (DSM‐5) (Guo et al., 2015); (2) being without electric convulsions, not taking antipsychotic drugs or sedatives 2 weeks before enrollment, and dextromanuality; (3) no obvious organic lesions based on head CT or MRI; (4) age ranged from 60 to 86 years old; and (5) informed consent signed by family members of patients. The exclusion criteria were as follows: (1) loss of consciousness caused by traumatic brain injury for more than 30 min, (2) a history of alcohol and neuroactive substance abuse, (3) mental retardation, (4) mental disorders caused by physical or cerebrovascular diseases, (5) liver and kidney dysfunction, (6) being with severe hearing impairment, and (7) being unwilling to participate in the study of patient's family. Based on the violence risk assessment (Wu, 2015), the violence was divided by the Broset Violence Checklist (BVC). There are six items in this checklist, which would be labeled as 0 or 1 point of each item. Patients with total points of 0 were divided into the low‐risk group, the ones with 1–2 points were divided into the medium‐risk group, and the ones with 3–6 points were divided into the high‐risk group. The total points of each patient were evaluated by two independent doctors two times per day for 3 days. Totally, 61, 102, and 145 patients were divided into high‐risk, medium‐risk group, and low‐risk groups, respectively. There were 28 male and 33 female patients in the high‐risk group with a mean age of 67.92 ± 8.63 (61–75) years. There were 48 male and 54 female patients in the medium‐risk group with a mean age of 69.96 ± 8.73 (62–79) years. There were 68 male and 77 female patients in the low‐risk group with a mean age of 71.87 ± 8.71 (62–84) years. Medical records including gender, age, marital status, education level, previous history of violence, family history, and complications of all the patients enrolled were collected and analyzed.

### 2.2 Detection and analysis of auditory P300

Auditory P300 was measured within 3 days after admission. Patients were instructed to wash their hair 1 day before the examination and the detection was carried out in a shielded soundproof room using the multi‐guide psychophysiological testing system produced by Shenzhen Brain Potential Industrial Development Co., LTD. Subjects should be seated and the installation of recording electrodes referred to the international 10/20 system electrode coordination method. Impedances were below 5k ohms and a silver chloride electrode with a diameter of 8 mm was used. Recording electrodes were affixed to Cz (noninverting) and reference electrodes were placed in both earlobes. In addition, ground electrodes were affixed to the middle of the forehead. The stimulus sequence was an odd‐ball paradigm consisting of a target stimulus and a nontarget stimulus with a ratio of 2:8. The target stimulus was randomly assigned to the nontarget stimulus. The target stimulus frequency was 2000 Hz and the sound intensity was 80 dB, while the nontarget stimulus was 1000 Hz and the sound was 60 dB. The duration of the above sound was 20 ms. The filtering range in the test is 1–30 Hz, and the scanning time is 800 ms. When the subjects mastered the essentials, the test would start, and 30–50 stacking would be conducted in each round of the test.

EPR P300 data were analyzed using the multichannel physiological and psychological detection and evaluation system (Acquisition and Analysis Subsystem, version V2.22) provided by Shenzhen Brain Potential Industrial Development Co., LTD. Before data analysis, wavelet denoising (15 dB), visually associated potential elimination (threshold ± 130μV), and baseline drift correction during stacking were carried out by using the built‐in functions of the system. Analysis indicators included (1) P300 amplitude: the distance from the maximum forward peak to the baseline between 250 and 550 ms after target stimulation, which was in the unit of microvolts (μV); (2) P300 latency: the straight‐line distance from the beginning of stimulation to the peak of P300 wave, which was in the unit of milliseconds (ms). It was performed for all patients every 3 days and five times in total.

### 2.3 Assessment of BVC

BVC is a short‐term risk assessment tool for violence (Wu, 2015). The scale has six items in total, including chaos, provocation, noise, verbal threat, wounding behavior, and destructive behavior. The scale assigns 0 to 1 point to each item according to the existence of the six previously discovered behaviors, and the maximum score of each item is 6 points. The higher the score, the higher the risk of aggressive behavior in the next 24 h. Low risk is 0, medium risk is 1–2 points, and high risk is 3–6 points. The subjects were evaluated by two professional psychiatrists trained in the scale, twice a day for three consecutive days.

### 2.4 Statistical analyses

Statistical analyses were performed using the SPSS V.20 software. All data were tested for normality and homogeneity of variance. Measurement data of normal distribution was presented as mean ± SDs. If not, measurement data were presented as median and quartiles. One‐way ANOVA or nonparametric Kruskal–Wallis *H* test was used for comparisons of measurement data among the three groups. The chi‐square test or Fisher's exact test was used for comparisons of counting data among the three groups. Univariate logistic regression analysis was used to analyze risk factors for severe violence of elderly schizophrenics followed by multivariate logistic regression analysis of severe violence of elderly schizophrenics. A *p*‐value of <.05 was considered statistically significant.

## 3 RESULTS

### 3.1 Comparisons of demographic data of patients among the three groups

There were no significant differences in gender, age, marital status, education level, disease duration, and complications among the three groups (all *p* > .05). As shown in Table 1, the incidence of a previous history of violence and delusion of persecution in the high‐risk group was significantly higher than that in the medium‐risk and low‐risk groups (all *p* < .05), while the incidence of regular medication and voluntary hospitalization in the high‐risk group was significantly lower (all *p* < .05). In addition, the incidence of a previous history of violence and delusion of persecution was significantly higher in the medium‐risk group than that in the low‐risk group (*p* < .05), while the incidence of regular medication and voluntary hospitalization in the medium‐risk group was significantly lower (*p* < .05).

**TABLE 1:** Comparative analysis of clinical characteristics of the three groups

Characteristics	High‐risk group (*n* = 61)	Medium‐risk group (*n* = 102)	Low‐risk group (*n* = 145)	*F*/*χ* ^2^ value	*p*
Gender (*n*)					
Male (%)	28 (45.90)	48 (47.06)	68 (46.90)	0.023	.989
Female (%)	33 (54.10)	54 (52.94)	77 (53.10)		
Age (years)	67.92 ± 8.63	69.96 ± 8.73	71.87 ± 8.71	0.096	.909
Marital status (*n*)					
Widowed (%)	10 (16.39)	16 (15.69)	23 (15.86)	0.526	2.971
Married (%)	49 (80.33)	84 (82.35)	117 (80.69)		
Unmarried (%)	2 (3.28)	2 (1.96)	5 (3.45)		
Education level (*n*)					
College degree or above (%)	10 (16.39)	23 (22.55)	26 (17.93)	1.331	.856
High school/Technical secondary school (%)	17 (27.87)	28 (27.45)	36 (24.83)		
Junior high and below (%)	34 (55.74)	54 (51.43)	83 (57.24)		
Disease course (years)	14.23 ± 3.09	13.67 ± 3.18	13.87 ± 3.21	0.251	.779
Previous history of violence	49 (80.33)^*#^	54 (52.943)^#^	64 (44.14)	10.832	.004
Complications (*n*)					
Hypertension (%)	7 (11.48)	9 (8.82)	9 (6.21)	1.699	.427
Diabetes (%)	8 (13.11)	7 (6.86)	10 (6.90)	2.912	.233
In work, *n* (%)	24 (39.34)	45 (44.12)	70 (48.28)	1.446	.485
Regular medication, *n* (%)	17 (27.87)^*#^	63 (61.76)	98 (67.59)	28.754	<.001
Voluntary hospitalization, *n* (%)	7 (11.48)^*#^	13 (28.43)^#^	64 (44.14)	39.322	<.001
Delusions of persecution, *n* (%)	39 (63.93)^*#^	36 (35.29)^#^	28 (19.31)	38.648	<.001

*Note*: *p* < .05 was considered as statistically significant.

^*^ *p* < .05 when compared with the medium‐risk group; ^#^ *p* < .05 when compared with the low‐risk group.

### 3.2 Varying P300 latency and amplitude in elderly schizophrenics with different levels of violence

As shown in Table 2, P300 latencies of the high‐risk group, medium‐risk group, and low‐risk group were 389.71 ± 34.91, 383.29 ± 36.19, and 376.28 ± 33.21 ms, respectively. The latency of P300 in the high‐risk group was higher than that in the low‐risk group (*p* < .05). There was no difference in P300 latency between the high‐risk and medium‐risk groups (*p* > .05). There was no difference in P300 latency between the medium‐risk and low‐risk groups (*p* > .05). P300 amplitudes of the three groups were 6.78 ± 2.13, 7.24 ± 1.98, and 7.81 ± 1.95 μV, respectively. The P300 amplitude of patients in the high‐risk group was significantly lower than those of the low‐risk group (*p* < .05). There was no difference in P300 amplitude between the high‐risk and medium‐risk groups (*p* > .05). There was no difference in P300 amplitude between the medium‐risk and low‐risk groups (*p* > .05).

**TABLE 2:** Comparative analysis of P300 latency and amplitude among the three groups

Parameters	High‐risk group (*n* = 61)	Medium‐risk group (*n* = 102)	Low‐risk group (*n* = 145)	*F* value	*p*
Latency (ms)	389.71 ± 34.91*	383.29 ± 36.19	376.28 ± 33.21	3.505	.031
Amplitude (μV)	6.78 ± 2.13*	7.24 ± 1.98	7.81 ± 1.95	6.315	.002

* *p* < .05 when compared with the low‐risk group.

### 3.3 Univariate logistic regression analysis of severe violence in elderly schizophrenics

There were significant differences in the previous history of violence, regular medication, voluntary hospitalization, delusion of persecution, and P300 latency and amplitude among the three groups, so these parameters were used as independent variables. Severe violence in elderly schizophrenics was used as the dependent variable. As shown in Table 3, the univariate logistic regression analysis showed that previous history of violence, delusion of persecution, and P300 latency and amplitude were independent factors for severe violence in elderly schizophrenics (odds ratio [OR]: 0.609, 95% confidence interval [CI]: 0.452–0.887, *p* = .003; OR: 0.118, 95% CI: 0.043–1.763, *p* < .001; OR: 3.201, 95% CI: 1.342–9.673, *p* = .016; and OR: 0.739, 95% CI: 0.601–0.967, *p =* .001, respectively).

**TABLE 3:** Univariate logistic regression analysis of elderly schizophrenics with severe violence

Variables	*β*	Wald *χ* ^2^	*p*	OR (95% CI)
Previous history of violence	–0.467	8.362	.003	0.609 (0.452–0.887)
Regular medication	1.421	5.611	.913	4.013 (1.282–12.098)
Voluntary hospitalization	0.654	1.867	.581	1.913 (0.821–3.187)
Delusions of persecution	–1.628	0.437	<.001	0.118 (0.043–1.763)
P300 latency	1.259	6.872	.016	3.201 (1.342–9.673)
P300 amplitude	–0.294	11.287	.001	0.739 (0.601–0.967)

*Note*: *p*<.05 was considered as statistically significant.

Abbreviation: CI, confidence interval.

### 3.4 Multivariate logistic regression analysis of severe violence in elderly schizophrenics

After adjusting for age, gender, and other confounding factors, previous history of violence, delusion of persecution, and P300 latency and amplitude were used as independent variables. Severe violence in elderly schizophrenics was used as the dependent variable. As shown in Table 4, the multivariate logistic regression analysis showed that previous history of violence, delusion of persecution, and P300 latency and amplitude were independent factors for severe violence in elderly schizophrenics (OR: 0.119, 95% CI: 0.013–0.307, *p* = .039; OR: 0.412, 95% CI: 0.231–0.742, *p* = .004; OR: 1.206, 95% CI: 1.041–1.621, *p* = .007; and OR: 0.367, 95% CI: 0.217–0.709, *p =* .001; respectively).

**TABLE 4:** Multivariate logistic regression analysis of elderly schizophrenics with severe violence

Variables	*β*	Wald *χ* ^2^	*p*	OR (95% CI)
Previous history of violence	–2.137	4.053	.039	0.119 (0.013–0.307)
Delusions of persecution	–0.881	8.562	.004	0.412 (0.231–0.742)
P300 latency	0.183	7.384	.007	1.206 (1.041–1.621)
P300 amplitude	–0.946	9.682	.001	0.367 (0.217–0.709)

*Note*: *p* < .05 was considered as statistically significant.

Abbreviation: CI, confidence interval.

## 4 DISCUSSION

Our study showed there were significant differences in previous history of violence, regular medication, voluntary admission, delusions of persecution, and ERP P300 parameters among elderly schizophrenics with different levels of violence. In addition, delusion of persecution, P300 latency, and P300 amplitude were independent factors associated with severe violence in elderly schizophrenics.

Schizophrenics are prone to behavioral and cognitive disorders and manifest as personality withdrawn, sensitive, poor logical ability. They thus cannot normally integrate into society, especially elderly schizophrenia patients, causing a serious impact on their quality of life (Driver et al., 2020). According to relevant statistics, about 40%−60% of schizophrenics have different degrees of cognitive dysfunction, and the degree of cognitive dysfunction is closely related to the chronic course of the disease and prognosis (Shi et al., 2021). Previous studies have pointed out that schizophrenics suffer from a wide range of cognitive impairments, including attention, alertness, motor memory, and executive ability (Peng et al., 2020; Yan et al., 2020)^.^ ERP P300 is a very valuable electrophysiological indicator to reflect cognitive impairment early, which can reflect the response, acceptance, and processing of subjects after receiving stimulation, and comprehensively reflect individual attention, perception, memory, judgment, and thinking ability (Jung et al., 2013; Philip & George, 2020; Sabeti et al., 2011). The decrease of P300 amplitude in schizophrenics indicates a decrease in the intensity of the brain's response to external stimuli. The latency mainly reflects the perception of stimulus information and the time needed to classify the stimulus. The longer latency of P300 in patients indicates that the neural processing speed of their brains decreases. Abhishek et al. (2018) pointed out that selective attention disorder in patients with schizophrenia may reduce the ability to distinguish and cause hallucinations and delusions. Due to the reduced allocation of attention resources and the reduced ability to classify and integrate information, the information stimuli received by patients make errors in information processing. It ultimately leads to the inability to distinguish the source of hallucinations of schizophrenics and makes schizophrenics attribute the perceived information to the external environment. Under the action of certain stimuli and psychological factors, patients with schizophrenia are easy to be in a state of anxiety, tension, and compulsion and misperceive outside information, leading to emotional overreaction followed by impulsive behavior (Brazil et al., 2012). The above mechanisms provide the theoretical basis for the violent behavior of schizophrenics under psychotic symptoms and psychological factors. Because P300 is an early indicator of cognitive dysfunction, it may be able to predict violent behavior.

The results of this study showed that the incidence of previous history of violence, regular medication, voluntary hospitalization, and delusions of persecution in the high‐risk group was significantly different from that in the medium‐risk and low‐risk groups. In addition, the incidence of previous history of violence, regular medication, voluntary hospitalization, and delusions of persecution in the medium‐risk group was significantly different from that in the low‐risk group. It is suggested that the higher the risk of violence, the higher the incidence of previous history of violence and delusion of persecution, and the lower the incidence of regular medication and voluntary hospitalization. The studies of Brazil et al. and Zukov et al. pointed out that aggressive behavior can lead to neuroelectrophysiological changes with reduced amplitude in both schizophrenia patients and normal people (Brazil et al., 2012; Zukov et al., 2008). Si Jianli et al. revealed that there was no significant difference in P300 latency between male schizophrenia patients with a high risk of aggression (BVC total score >2 points) and those with no risk of aggression (BVC total score ≤2 points), but the amplitude was significantly reduced (Si & Yuan, 2020). Our study showed that the latency and amplitude of P300 in the high‐risk group were significantly higher than those in the low‐risk group. Consistent with the above research results, the P300 latency has a tendency to prolong with the increase of the level of risk of violence in elderly schizophrenics. The lack of statistical difference in latency between the two groups in their study may be related to the threshold selection of the BVC risk scale assessment. Jeon et al. pointed out that BVC > 2 was more appropriate as the threshold of high violence tendency, while Lockertsen et al. believed that BVC > 4 was more appropriate as the threshold of high violence tendency (Jeon & Polich, 2003; Lockertsen et al., 2021). Different values of the threshold of high violence tendency may lead to inaccurate judgment of violence risk and thus affect the judgment of P300 latency and amplitude change.

In order to further analyze risk factors of violence of elderly schizophrenics in the short term, we performed analyses. Results of univariate logistic regression analysis demonstrated that previous history of violence, delusion of persecution, and P300 latency and amplitude were associated with severe violence in elderly schizophrenics. After correction of relevant influencing factors, multivariate logistic regression analysis showed that delusion of persecution, P300 latency, and P300 amplitude were risk factors of severe violence in elderly schizophrenics. In conclusion, the latency and amplitude of P300 are reliable tools for predicting violent behavior. In view of the advantages of P300, such as low price, short time consumption, easy‐to‐obtain results, and simple operation, it is particularly important to conduct a regular evaluation for schizophrenic patients. In addition, it may be more important for clinicians to assess the risk of violence before discharge. In some cases, patients may lie, cover up, cheat, and take other measures to achieve the purpose of discharge. These behaviors will affect the judgment of physicians, and the inclusion of P300 as a reference can improve the accuracy of clinical judgment (Zhang et al., 2012).

However, this study also had some limitations as follows: (1) The observation time of this study was short, and the situational stressors and patients’ emotional state were not included in the study. (2) The subjects of this study were only elderly schizophrenic patients, and the sample size was small. (3) This was a single‐cohort study without a control group. Thus, all results should be interpreted cautiously. The sample size should be expanded later to explore the strong evidence that affects the occurrence of violent behavior in schizophrenic patients and then build the prediction model of violent behavior in schizophrenic patients.

## 5 CONCLUSION

Our study showed that the latency and amplitude of ERP P300 in elderly schizophrenics with different levels of violence were significantly different. P300 latency and amplitude were independent risk factors for severe violence in elderly schizophrenics, suggesting that P300 could be used to predict the progression of elderly schizophrenics.

## AUTHOR CONTRIBUTIONS

Zhenguo Wu, Zixuan Zhou, and Guanli Su contributed to the conception and design of the study and wrote the manuscript. Wenting Lu, Lin Liu, and Qifeng Zhu performed the experiments and collected and analyzed the data. All authors reviewed and approved the final version of the manuscript.

## FUNDING INFORMATION

No funding was received for this study.

## CONFLICT OF INTEREST STATEMENT

The authors declare no conflicts of interest.

### PEER REVIEW

The peer review history for this article is available at https://publons.com/publon/10.1002/brb3.2966.

## Data Availability

The data sets generated and analyzed during the current study are available from the corresponding author on reasonable request.
